# *trans*-Bis(dimethyl sulfoxide-κ*O*)bis­(thio­semicarbazide-κ^2^ *N* ^1^,*S*)cadmium dipicrate dihydrate

**DOI:** 10.1107/S1600536810053602

**Published:** 2010-12-24

**Authors:** R. Shanthakumari, R. Hema, K. Ramamurthy, Helen Stoeckli-Evans

**Affiliations:** a Department of Physics, Government Arts College for Women, Pudukkottai 622 001, India; b Department of Physics, Seethalakshmi Ramaswami College (Autonomous), Tiruchirappalli 620 002, India; c Crystal Growth and Thin Film Laboratory, School of Physics, Bharathidasan University, Tiruchirapalli 620 024, India; d Institute of Physics, University of Neuchâtel, Rue Emile-Argand 11, CH-2009 Neuchâtel, Switzerland

## Abstract

In the cation of the title compound, [Cd(CH_5_N_3_S)_2_(C_2_H_6_OS)_2_](C_6_H_2_N_3_O_7_)_2_·2H_2_O, the Cd^II^ atom is located on an inversion center. It is hexa­coordinated in an octahedral fashion by two thio­semicarbazide mol­ecules, which coordinate in a bidentate manner *via* the S and N atoms, and to the O atom of two dimethyl sufoxide (DMSO) mol­ecules. The charges are equilibrated by two picrate anions and the complex crystallizes as a dihydrate. In the crystal, these units are linked by a number of O—H⋯O and N—H⋯S hydrogen bonds and weak C—H⋯O inter­actions, forming a three-dimensional network.

## Related literature

For the role of hydrogen bonding in the construction of supra­molecular structures, see: Braga *et al.* (2004 ▶). For the crystal structure of a similar compound, see: Li *et al.* (2006 ▶).
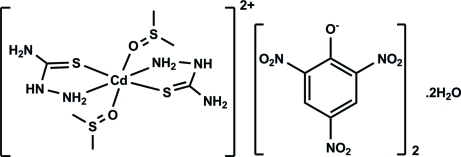


## Experimental

### 

#### Crystal data

[Cd(CH_5_N_3_S)_2_(C_2_H_6_OS)_2_](C_6_H_2_N_3_O_7_)_2_·2H_2_O
*M* *_r_* = 943.18
Triclinic, 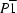

*a* = 5.3496 (3) Å
*b* = 11.0788 (6) Å
*c* = 15.0049 (8) Å
α = 98.745 (5)°
β = 98.288 (4)°
γ = 95.032 (5)°
*V* = 864.30 (8) Å^3^
*Z* = 1
Mo *K*α radiation
μ = 0.97 mm^−1^
*T* = 173 K
0.45 × 0.30 × 0.21 mm

#### Data collection

STOE IPDS 2 diffractometer
Absorption correction: multi-scan [*MULscanABS* in *PLATON* (Spek, 2009 ▶)] *T*_min_ = 0.773, *T*_max_ = 1.000
10206 measured reflections
3273 independent reflections
3058 reflections with *I* > 2σ(*I*)
*R*_int_ = 0.037

#### Refinement

*R*[*F* ^2^ > 2σ(*F* ^2^)] = 0.023
*wR*(*F* ^2^) = 0.059
*S* = 1.05
3273 reflections
272 parameters
H atoms treated by a mixture of independent and constrained refinement
Δρ_max_ = 0.45 e Å^−3^
Δρ_min_ = −0.61 e Å^−3^

### 

Data collection: *X-AREA* (Stoe & Cie, 2009 ▶); cell refinement: *X-AREA*; data reduction: *X-RED32* (Stoe & Cie, 2009 ▶); program(s) used to solve structure: *SHELXS97* (Sheldrick, 2008 ▶); program(s) used to refine structure: *SHELXL97* (Sheldrick, 2008 ▶); molecular graphics: *PLATON* (Spek, 2009 ▶); software used to prepare material for publication: *SHELXL97* and *PLATON* (Spek, 2009 ▶).

## Supplementary Material

Crystal structure: contains datablocks I, global. DOI: 10.1107/S1600536810053602/hg2776sup1.cif

Structure factors: contains datablocks I. DOI: 10.1107/S1600536810053602/hg2776Isup2.hkl

Additional supplementary materials:  crystallographic information; 3D view; checkCIF report

## Figures and Tables

**Table 1:** Hydrogen-bond geometry (Å, °)

*D*—H⋯*A*	*D*—H	H⋯*A*	*D*⋯*A*	*D*—H⋯*A*
N1—H1*NA*⋯O2^i^	0.87 (3)	2.00 (3)	2.773 (3)	148 (2)
N1—H1*NA*⋯O8^i^	0.87 (3)	2.47 (2)	3.178 (2)	140 (2)
N1—H1*NB*⋯O7^ii^	0.79 (3)	2.25 (3)	3.037 (3)	175 (2)
N2—H2*N*⋯O2^i^	0.88 (3)	1.97 (3)	2.755 (2)	148 (2)
N2—H2*N*⋯O3^i^	0.88 (3)	2.33 (3)	3.026 (2)	136 (2)
N3—H3*NA*⋯S1^i^	0.83 (3)	2.69 (3)	3.4653 (17)	155 (2)
N3—H3*NB*⋯O1*W*^i^	0.86 (2)	2.21 (2)	3.049 (3)	165 (2)
O1*W*—H1*WA*⋯O5^iii^	0.76 (4)	2.46 (3)	3.081 (2)	140 (3)
O1*W*—H1*WB*⋯O4^iv^	0.84 (3)	2.03 (3)	2.844 (2)	165 (3)
C3—H3*A*⋯O1*W*	0.98	2.60	3.272 (3)	126

Symmetry codes: (i) 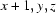; (ii) 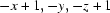; (iii) 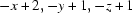; (iv) 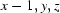.

**Fig. 1.**
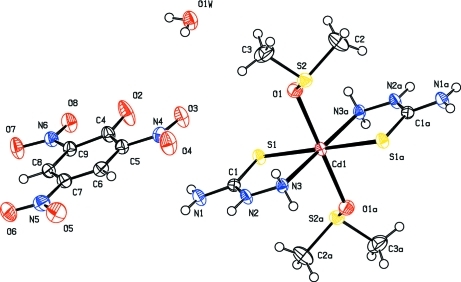
View of the molecular structure of the title compound, showing the atom-labelling scheme. Displacement ellipsoids are drawn at the 50% probability level [symmetry code: (a) = -x + 2, -y, -z]. The symmetry related picrate anion and water molecule of crystallization are not shown.

**Fig. 2.**
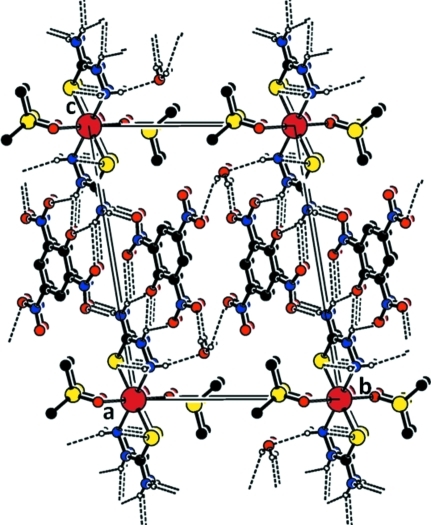
A view along the a axis of the crystal packing of the title compound. The hydrogen bonds are shown as dashed lines (see Table 1 for details; C-bound H-atoms not involved in C—H···O interactions have been omitted for clarity].

## supplementary crystallographic information

### Comment

Intermolecular and inter-ionic hydrogen bonding interactions, which are not only the strongest of the noncovalent interactions but also highly directional, play an important role in constructing supramolecular structures (Braga *et al.*, 2004). Recently we have obtained crystals of the title compound from the reaction of thiosemicarbazide, picric acid and DMSO, and we report herein on its crystal structure.

In the cation of the title compound the cadmium(II) atom is hexa-coordinated to two thiosemcarbazide and to two DMSO molecules (Fig. 1). This cation is centrosymmetric with the metal atom being located on an invesion center. The thiosemicarbazide molecules behave as bidentate ligands coordinating *via* atoms N3 and S1. The bond distances and angles are similar to those reported for a similar complex, bis(Thiosemicarbazide)-diaqua-cadmium(II) bis(maleate) dihydrate (Li *et al.*, 2006).

In the crystal the cation, the picrate anions and the water molecules of crystallization are involved in N—H···O, O—H···O and N—H···S hydrogen bonds and C—H···O interactions, to form a three-dimensional supramolecular network (Table 1 and Fig. 2).

### Experimental

The title compound was synthesized by reacting thiosemicarbazide, picric acid and cadmium bromide in a 2:1:1 (1.8 g: 2.5 g: 2.8 g) molar ratio. The calculated amount of thiosemicarbazide and cadmium bromide were dissolved in distilled water and the required amount of picric acid (dissolved in acetone) was added to the solution slowly with stirring. Within a few minutes the solution became turbid. The reaction was ensured with continous stirring. After 1 h a yellow salt was deposited at the bottom of the beaker and it was filtered off and dried. This solid was recrystallized in a DMSO solution to obtain yellow rod-like crystals of the title compound on slow evaporation of the solvent.

### Refinement

The water molecule, the NH_2_ and NH H-atoms were located in difference Fourier maps and were freely refined. The C-bound H atoms were included in calculated positions and treated as riding atoms; C—H = 0.95 Å and 0.98 Å for CH and methyl H-atoms, respectively, with *U*_iso_(H) = k × *U*_eq_(C), where k = 1.2 for CH H-atoms, and k = 1.5 for methyl H-atoms.

### Crystal data

**Table:**

[Cd(CH_5_N_3_S)_2_(C_2_H_6_OS)_2_](C_6_H_2_N_3_O_7_)_2_·2H_2_O	*Z* = 1
*M**_r_* = 943.18	*F*(000) = 478
Triclinic, *P*1	*D*_x_ = 1.812 Mg m^−^^3^
Hall symbol: -P 1	Mo *K*α radiation, λ = 0.71073 Å
*a* = 5.3496 (3) Å	Cell parameters from 15389 reflections
*b* = 11.0788 (6) Å	θ = 1.4–26.2°
*c* = 15.0049 (8) Å	µ = 0.97 mm^−^^1^
α = 98.745 (5)°	*T* = 173 K
β = 98.288 (4)°	Rod, yellow
γ = 95.032 (5)°	0.45 × 0.30 × 0.21 mm
*V* = 864.30 (8) Å^3^	

### Data collection

**Table:**

STOE IPDS 2 diffractometer	3273 independent reflections
Radiation source: fine-focus sealed tube	3058 reflections with *I* > 2σ(*I*)
graphite	*R*_int_ = 0.037
φ + ω scans	θ_max_ = 25.6°, θ_min_ = 1.4°
Absorption correction: multi-scan [*MULscanABS* in *PLATON* (Spek, 2009)]	*h* = −6→6
*T*_min_ = 0.773, *T*_max_ = 1.000	*k* = −13→13
10206 measured reflections	*l* = −18→18

### Refinement

**Table:**

Refinement on *F*^2^	Secondary atom site location: difference Fourier map
Least-squares matrix: full	Hydrogen site location: inferred from neighbouring sites
*R*[*F*^2^ > 2σ(*F*^2^)] = 0.023	H atoms treated by a mixture of independent and constrained refinement
*wR*(*F*^2^) = 0.059	*w* = 1/[σ^2^(*F*_o_^2^) + (0.0346*P*)^2^ + 0.3178*P*] where *P* = (*F*_o_^2^ + 2*F*_c_^2^)/3
*S* = 1.05	(Δ/σ)_max_ < 0.001
3273 reflections	Δρ_max_ = 0.45 e Å^−^^3^
272 parameters	Δρ_min_ = −0.61 e Å^−^^3^
0 restraints	Extinction correction: *SHELXL*, Fc^*^=kFc[1+0.001xFc^2^λ^3^/sin(2θ)]^-1/4^
Primary atom site location: structure-invariant direct methods	Extinction coefficient: 0.0043 (12)

### Special details

**Table:**

Geometry. Bond distances, angles *etc*. have been calculated using the rounded fractional coordinates. All su's are estimated from the variances of the (full) variance-covariance matrix. The cell e.s.d.'s are taken into account in the estimation of distances, angles and torsion angles
Refinement. Refinement of *F*^2^ against ALL reflections. The weighted *R*-factor *wR* and goodness of fit *S* are based on *F*^2^, conventional *R*-factors *R* are based on *F*, with *F* set to zero for negative *F*^2^. The threshold expression of *F*^2^ > σ(*F*^2^) is used only for calculating *R*-factors(gt) *etc*. and is not relevant to the choice of reflections for refinement. *R*-factors based on *F*^2^ are statistically about twice as large as those based on *F*, and *R*- factors based on ALL data will be even larger.

### Fractional atomic coordinates and isotropic or equivalent isotropic displacement parameters (Å^2^)

**Table:**

	*x*	*y*	*z*	*U*_iso_*/*U*_eq_	
Cd1	1.00000	0.00000	0.00000	0.0212 (1)	
S1	0.82169 (8)	−0.05948 (4)	0.13804 (3)	0.0235 (1)	
S2	0.86010 (9)	0.28748 (4)	−0.02021 (3)	0.0269 (1)	
O1	0.7484 (3)	0.16934 (13)	0.00566 (10)	0.0311 (4)	
N1	1.0413 (4)	0.00580 (18)	0.30857 (11)	0.0278 (5)	
N2	1.2618 (3)	0.08648 (15)	0.20958 (11)	0.0238 (4)	
N3	1.3070 (3)	0.10988 (16)	0.12296 (10)	0.0217 (5)	
C1	1.0580 (3)	0.01743 (16)	0.22259 (12)	0.0205 (5)	
C2	0.6902 (5)	0.2973 (2)	−0.12943 (16)	0.0415 (7)	
C3	0.7285 (5)	0.4052 (2)	0.04653 (19)	0.0450 (8)	
O2	0.4733 (3)	0.16250 (19)	0.39020 (11)	0.0518 (6)	
O3	0.7565 (3)	0.24983 (16)	0.27887 (10)	0.0392 (5)	
O4	1.0803 (3)	0.37978 (17)	0.33787 (11)	0.0464 (6)	
O5	1.4020 (3)	0.46927 (16)	0.64927 (12)	0.0458 (5)	
O6	1.1907 (3)	0.40466 (16)	0.74913 (11)	0.0441 (5)	
O7	0.4239 (3)	0.14920 (16)	0.65922 (10)	0.0406 (5)	
O8	0.2735 (3)	0.07080 (14)	0.51955 (10)	0.0345 (4)	
N4	0.9009 (3)	0.30544 (15)	0.34539 (11)	0.0273 (5)	
N5	1.2187 (3)	0.40910 (17)	0.66954 (12)	0.0340 (5)	
N6	0.4329 (3)	0.13765 (15)	0.57626 (11)	0.0259 (5)	
C4	0.6439 (4)	0.21409 (19)	0.45209 (13)	0.0262 (5)	
C5	0.8669 (4)	0.28941 (17)	0.43761 (13)	0.0243 (5)	
C6	1.0516 (4)	0.35091 (17)	0.50671 (13)	0.0259 (5)	
C7	1.0285 (4)	0.34163 (18)	0.59632 (13)	0.0262 (5)	
C8	0.8251 (4)	0.27133 (18)	0.61769 (13)	0.0253 (5)	
C9	0.6418 (4)	0.20911 (17)	0.54848 (13)	0.0241 (5)	
O1W	0.2815 (4)	0.38530 (16)	0.17293 (11)	0.0367 (5)	
H1NA	1.152 (5)	0.048 (2)	0.3525 (17)	0.030 (6)*	
H1NB	0.915 (5)	−0.031 (2)	0.3166 (16)	0.028 (6)*	
H2A	0.72150	0.22870	−0.17440	0.0620*	
H2B	0.74760	0.37520	−0.14750	0.0620*	
H2C	0.50790	0.29350	−0.12660	0.0620*	
H2N	1.374 (5)	0.123 (2)	0.2565 (18)	0.036 (6)*	
H3NA	1.449 (5)	0.087 (2)	0.1177 (16)	0.030 (6)*	
H3A	0.54440	0.38420	0.04030	0.0680*	
H3B	0.76360	0.48340	0.02520	0.0680*	
H3C	0.80520	0.41300	0.11080	0.0680*	
H3NB	1.311 (4)	0.188 (2)	0.1279 (15)	0.026 (6)*	
H6	1.19300	0.39900	0.49330	0.0310*	
H8	0.81260	0.26630	0.67960	0.0300*	
H1WA	0.403 (7)	0.425 (3)	0.197 (2)	0.056 (10)*	
H1WB	0.197 (7)	0.377 (3)	0.215 (2)	0.065 (10)*	

### Atomic displacement parameters (Å^2^)

**Table:**

	*U*^11^	*U*^22^	*U*^33^	*U*^12^	*U*^13^	*U*^23^
Cd1	0.0240 (1)	0.0238 (1)	0.0150 (1)	0.0017 (1)	0.0020 (1)	0.0022 (1)
S1	0.0209 (2)	0.0285 (2)	0.0186 (2)	−0.0053 (2)	0.0014 (2)	0.0027 (2)
S2	0.0252 (2)	0.0271 (2)	0.0270 (2)	0.0032 (2)	0.0013 (2)	0.0032 (2)
O1	0.0370 (8)	0.0301 (7)	0.0314 (7)	0.0111 (6)	0.0105 (6)	0.0123 (6)
N1	0.0249 (9)	0.0385 (9)	0.0180 (8)	−0.0057 (8)	0.0014 (7)	0.0058 (7)
N2	0.0218 (8)	0.0327 (8)	0.0144 (7)	−0.0043 (7)	0.0000 (6)	0.0027 (6)
N3	0.0199 (8)	0.0262 (9)	0.0189 (8)	−0.0015 (7)	0.0052 (6)	0.0035 (6)
C1	0.0186 (8)	0.0223 (9)	0.0200 (9)	0.0019 (7)	0.0027 (7)	0.0025 (7)
C2	0.0473 (13)	0.0404 (12)	0.0349 (12)	−0.0064 (10)	−0.0060 (10)	0.0184 (10)
C3	0.0392 (12)	0.0376 (12)	0.0556 (15)	0.0126 (10)	0.0067 (11)	−0.0044 (11)
O2	0.0445 (10)	0.0797 (13)	0.0210 (7)	−0.0336 (9)	−0.0026 (7)	0.0068 (8)
O3	0.0430 (9)	0.0490 (9)	0.0216 (7)	−0.0094 (7)	0.0035 (6)	0.0032 (7)
O4	0.0449 (10)	0.0565 (10)	0.0356 (9)	−0.0181 (8)	0.0134 (7)	0.0075 (7)
O5	0.0294 (8)	0.0468 (10)	0.0503 (10)	−0.0123 (7)	−0.0025 (7)	−0.0086 (8)
O6	0.0443 (9)	0.0511 (10)	0.0272 (8)	−0.0005 (8)	−0.0069 (7)	−0.0083 (7)
O7	0.0405 (9)	0.0577 (10)	0.0211 (7)	−0.0112 (8)	0.0084 (6)	0.0049 (7)
O8	0.0306 (8)	0.0407 (8)	0.0278 (7)	−0.0114 (6)	0.0032 (6)	0.0018 (6)
N4	0.0281 (8)	0.0279 (8)	0.0267 (9)	0.0018 (7)	0.0080 (7)	0.0048 (7)
N5	0.0273 (9)	0.0330 (9)	0.0341 (10)	0.0002 (7)	−0.0052 (7)	−0.0072 (8)
N6	0.0240 (8)	0.0302 (8)	0.0225 (8)	−0.0015 (7)	0.0032 (7)	0.0040 (6)
C4	0.0243 (9)	0.0311 (10)	0.0202 (9)	−0.0043 (8)	0.0014 (7)	0.0016 (7)
C5	0.0248 (9)	0.0253 (9)	0.0221 (9)	0.0013 (7)	0.0037 (7)	0.0026 (7)
C6	0.0221 (9)	0.0233 (9)	0.0305 (10)	−0.0007 (7)	0.0039 (8)	0.0008 (8)
C7	0.0234 (9)	0.0254 (9)	0.0253 (9)	−0.0001 (8)	−0.0020 (7)	−0.0025 (7)
C8	0.0251 (10)	0.0280 (9)	0.0204 (9)	0.0022 (8)	0.0001 (7)	−0.0001 (7)
C9	0.0235 (9)	0.0254 (9)	0.0221 (9)	−0.0009 (7)	0.0040 (7)	0.0019 (7)
O1W	0.0375 (9)	0.0432 (9)	0.0254 (8)	−0.0032 (7)	0.0030 (7)	−0.0006 (7)

### Geometric parameters (Å, °)

**Table:**

Cd1—S1	2.5512 (5)	N1—H1NB	0.79 (3)
Cd1—O1	2.4013 (15)	N1—H1NA	0.87 (3)
Cd1—N3	2.3824 (16)	N2—H2N	0.88 (3)
Cd1—S1^i^	2.5512 (5)	N3—H3NB	0.86 (2)
Cd1—O1^i^	2.4013 (15)	N3—H3NA	0.83 (3)
Cd1—N3^i^	2.3824 (16)	N4—C5	1.456 (3)
S1—C1	1.7186 (18)	N5—C7	1.448 (3)
S2—O1	1.5182 (15)	N6—C9	1.459 (3)
S2—C2	1.779 (2)	C2—H2C	0.9800
S2—C3	1.780 (3)	C2—H2A	0.9800
O2—C4	1.235 (3)	C2—H2B	0.9800
O3—N4	1.217 (2)	C3—H3C	0.9800
O4—N4	1.238 (2)	C3—H3A	0.9800
O5—N5	1.238 (2)	C3—H3B	0.9800
O6—N5	1.233 (2)	C4—C5	1.457 (3)
O7—N6	1.240 (2)	C4—C9	1.457 (3)
O8—N6	1.220 (2)	C5—C6	1.374 (3)
O1W—H1WA	0.76 (4)	C6—C7	1.386 (3)
O1W—H1WB	0.84 (3)	C7—C8	1.386 (3)
N1—C1	1.331 (2)	C8—C9	1.373 (3)
N2—N3	1.413 (2)	C6—H6	0.9500
N2—C1	1.331 (2)	C8—H8	0.9500
			
S1—Cd1—O1	88.75 (4)	O5—N5—C7	118.35 (17)
S1—Cd1—N3	78.48 (4)	O7—N6—C9	117.15 (16)
S1—Cd1—S1^i^	180.00	O8—N6—C9	120.80 (16)
S1—Cd1—O1^i^	91.25 (4)	O7—N6—O8	122.04 (17)
S1—Cd1—N3^i^	101.53 (4)	S1—C1—N1	118.01 (14)
O1—Cd1—N3	91.01 (6)	S1—C1—N2	125.54 (14)
S1^i^—Cd1—O1	91.25 (4)	N1—C1—N2	116.43 (17)
O1—Cd1—O1^i^	180.00	S2—C2—H2C	110.00
O1—Cd1—N3^i^	88.99 (6)	H2A—C2—H2B	109.00
S1^i^—Cd1—N3	101.53 (4)	S2—C2—H2B	109.00
O1^i^—Cd1—N3	88.99 (6)	H2B—C2—H2C	109.00
N3—Cd1—N3^i^	180.00	S2—C2—H2A	109.00
S1^i^—Cd1—O1^i^	88.75 (4)	H2A—C2—H2C	109.00
S1^i^—Cd1—N3^i^	78.48 (4)	S2—C3—H3B	109.00
O1^i^—Cd1—N3^i^	91.01 (6)	H3A—C3—H3C	110.00
Cd1—S1—C1	98.67 (6)	H3B—C3—H3C	109.00
O1—S2—C2	106.27 (10)	H3A—C3—H3B	109.00
O1—S2—C3	104.19 (10)	S2—C3—H3A	109.00
C2—S2—C3	98.64 (12)	S2—C3—H3C	110.00
Cd1—O1—S2	117.54 (9)	O2—C4—C9	123.8 (2)
H1WA—O1W—H1WB	104 (3)	C5—C4—C9	112.18 (17)
N3—N2—C1	123.73 (16)	O2—C4—C5	124.06 (18)
Cd1—N3—N2	113.40 (11)	N4—C5—C6	115.94 (18)
C1—N1—H1NB	116.3 (17)	C4—C5—C6	124.10 (18)
H1NA—N1—H1NB	123 (2)	N4—C5—C4	119.95 (17)
C1—N1—H1NA	119.5 (16)	C5—C6—C7	118.96 (19)
N3—N2—H2N	116.2 (17)	N5—C7—C6	119.19 (18)
C1—N2—H2N	120.0 (17)	C6—C7—C8	121.65 (19)
Cd1—N3—H3NB	113.7 (15)	N5—C7—C8	119.15 (17)
Cd1—N3—H3NA	109.2 (16)	C7—C8—C9	119.29 (18)
N2—N3—H3NB	103.6 (15)	N6—C9—C8	116.16 (17)
H3NA—N3—H3NB	112 (2)	C4—C9—C8	123.79 (19)
N2—N3—H3NA	105.1 (16)	N6—C9—C4	120.03 (17)
O3—N4—O4	121.57 (17)	C5—C6—H6	121.00
O4—N4—C5	117.04 (16)	C7—C6—H6	121.00
O3—N4—C5	121.38 (17)	C7—C8—H8	120.00
O6—N5—C7	118.75 (17)	C9—C8—H8	120.00
O5—N5—O6	122.90 (18)		
			
O1—Cd1—S1—C1	−93.63 (7)	O5—N5—C7—C8	178.44 (19)
N3—Cd1—S1—C1	−2.35 (7)	O6—N5—C7—C6	176.82 (19)
O1^i^—Cd1—S1—C1	86.37 (7)	O6—N5—C7—C8	−1.9 (3)
N3^i^—Cd1—S1—C1	177.65 (7)	O7—N6—C9—C4	−171.03 (18)
S1—Cd1—O1—S2	145.05 (8)	O7—N6—C9—C8	7.4 (3)
N3—Cd1—O1—S2	66.61 (9)	O8—N6—C9—C4	8.2 (3)
S1^i^—Cd1—O1—S2	−34.95 (8)	O8—N6—C9—C8	−173.45 (18)
N3^i^—Cd1—O1—S2	−113.40 (9)	O2—C4—C5—N4	−1.2 (3)
S1—Cd1—N3—N2	3.88 (11)	O2—C4—C5—C6	177.2 (2)
O1—Cd1—N3—N2	92.39 (12)	C9—C4—C5—N4	−179.76 (17)
S1^i^—Cd1—N3—N2	−176.13 (11)	C9—C4—C5—C6	−1.3 (3)
O1^i^—Cd1—N3—N2	−87.61 (12)	O2—C4—C9—N6	1.6 (3)
Cd1—S1—C1—N1	−177.43 (15)	O2—C4—C9—C8	−176.7 (2)
Cd1—S1—C1—N2	1.06 (17)	C5—C4—C9—N6	−179.89 (17)
C2—S2—O1—Cd1	106.89 (11)	C5—C4—C9—C8	1.9 (3)
C3—S2—O1—Cd1	−149.51 (11)	N4—C5—C6—C7	178.76 (18)
C1—N2—N3—Cd1	−4.9 (2)	C4—C5—C6—C7	0.2 (3)
N3—N2—C1—S1	2.5 (3)	C5—C6—C7—N5	−178.22 (18)
N3—N2—C1—N1	−178.99 (17)	C5—C6—C7—C8	0.5 (3)
O3—N4—C5—C4	−5.6 (3)	N5—C7—C8—C9	178.76 (19)
O3—N4—C5—C6	175.84 (19)	C6—C7—C8—C9	0.1 (3)
O4—N4—C5—C4	173.14 (18)	C7—C8—C9—N6	−179.67 (18)
O4—N4—C5—C6	−5.5 (3)	C7—C8—C9—C4	−1.3 (3)
O5—N5—C7—C6	−2.9 (3)		

Symmetry codes: (i) −*x*+2, −*y*, −*z*.

### Hydrogen-bond geometry (Å, °)

**Table:**

*D*—H···*A*	*D*—H	H···*A*	*D*···*A*	*D*—H···*A*
N1—H1NA···O2^ii^	0.87 (3)	2.00 (3)	2.773 (3)	148 (2)
N1—H1NA···O8^ii^	0.87 (3)	2.47 (2)	3.178 (2)	140 (2)
N1—H1NB···O7^iii^	0.79 (3)	2.25 (3)	3.037 (3)	175 (2)
N2—H2N···O2^ii^	0.88 (3)	1.97 (3)	2.755 (2)	148 (2)
N2—H2N···O3^ii^	0.88 (3)	2.33 (3)	3.026 (2)	136 (2)
N3—H3NA···S1^ii^	0.83 (3)	2.69 (3)	3.4653 (17)	155 (2)
N3—H3NB···O1W^ii^	0.86 (2)	2.21 (2)	3.049 (3)	165 (2)
O1W—H1WA···O5^iv^	0.76 (4)	2.46 (3)	3.081 (2)	140 (3)
O1W—H1WB···O4^v^	0.84 (3)	2.03 (3)	2.844 (2)	165 (3)
C3—H3A···O1W	0.98	2.60	3.272 (3)	126
C6—H6···O4	0.95	2.30	2.626 (3)	100
C8—H8···O7	0.95	2.31	2.638 (3)	100

Symmetry codes: (ii) *x*+1, *y*, *z*; (iii) −*x*+1, −*y*, −*z*+1; (iv) −*x*+2, −*y*+1, −*z*+1; (v) *x*−1, *y*, *z*.
